# Severe Fournier’s gangrene in a patient with rectal cancer: case report and literature review

**DOI:** 10.1186/s12957-016-0989-z

**Published:** 2016-09-01

**Authors:** Yu Yoshino, Kimihiko Funahashi, Rei Okada, Yasuyuki Miura, Takayuki Suzuki, Takamaru Koda, Kimihiko Yoshida, Junichi Koike, Hiroyuki Shiokawa, Mitsunori Ushigome, Tomoaki Kaneko, Yasuo Nagashima, Mayu Goto, Akiharu Kurihara, Hironori Kaneko

**Affiliations:** Department of General and Gastroenterological Surgery, Toho University Omori Medical Center, 6-11-1 Omorinishi Ota-Ku, Tokyo, 143-8541 Japan

**Keywords:** Fournier’s gangrene, Rectal cancer, Surgical treatment, Reconstructive surgery

## Abstract

**Background:**

Fournier’s gangrene in the setting of rectal cancer is rare. Treatment for Fournier’s gangrene associated with rectal cancer is more complex than other cases of Fournier’s gangrene. We report on a patient with severe Fournier’s gangrene in the setting of locally advanced rectal cancer who was treated with a combined modality therapy.

**Case presentation:**

A 65-year-old man presented with general fatigue and anal pain. The medical and surgical histories were unremarkable. A black spot on the perineal skin surrounded by erythema was found on physical examination, suspicious for Fournier’s gangrene. Computed tomography scan showed a rectal tumor invading into the bladder (clinically T4bN2M0) and abscess formation with emphysema around the rectum. He was thus diagnosed with locally advanced rectal cancer and Fournier’s gangrene with a severity index score of 12 points. We created a diverting loop colostomy of the transverse colon and performed extensive debridement of the perineum and perianal area. Fifty days later, the patient underwent radical total pelvic exenteration with sacrectomy. In addition, reconstruction of the soft tissue defect was performed using the rectus muscle, the gluteus maximus muscle, and the femoral muscle. Histopathological findings of the specimen were as follows: the tumor was a moderately adenocarcinoma with invasion to the bladder and the prostate (T4b), metastases to four resected lymph nodes (N2), and lymphovascular invasion. There were no major postoperative complications, and the patient was discharged 108 days postoperatively.

**Conclusions:**

We report a rare case of locally invasive rectal cancer associated with Fournier’s gangrene. This case highlights a usual cause of Fournier’s gangrene. Physicians should be cognizant not only of the more common condition but also of the rare presentations including those associated with rectal cancer.

## Background

Fournier’s gangrene (FG) is necrotizing fasciitis and commonly begins without trauma or urinary tract disease. FG in the setting of rectal cancer is rare. A specific risk factor is rectal cancer perforation. Treatment includes extensive debridement of the areas of necrosis and the administration of broad-spectrum intravenous antibiotics. In the setting of rectal cancer, the causative rectal tumor should be removed, but the timing of this is a complex clinical decision.

We report on a patient with severe FG in the setting of locally advanced rectal cancer who was treated with a combined modality therapy.

## Case presentation

**Fig. 1:**
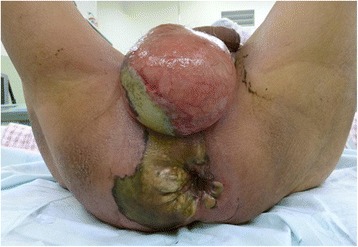
The appearance of the patient’s perineum. A black spot and emphysema were found in the perineal skin

**Fig. 2:**
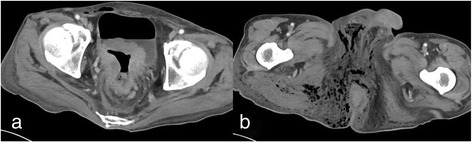
Computed tomography scan showing a rectal tumor invading the bladder (**a**) and abscess formation with emphysema in the pelvis and perineum (**b**)

**Fig. 3:**
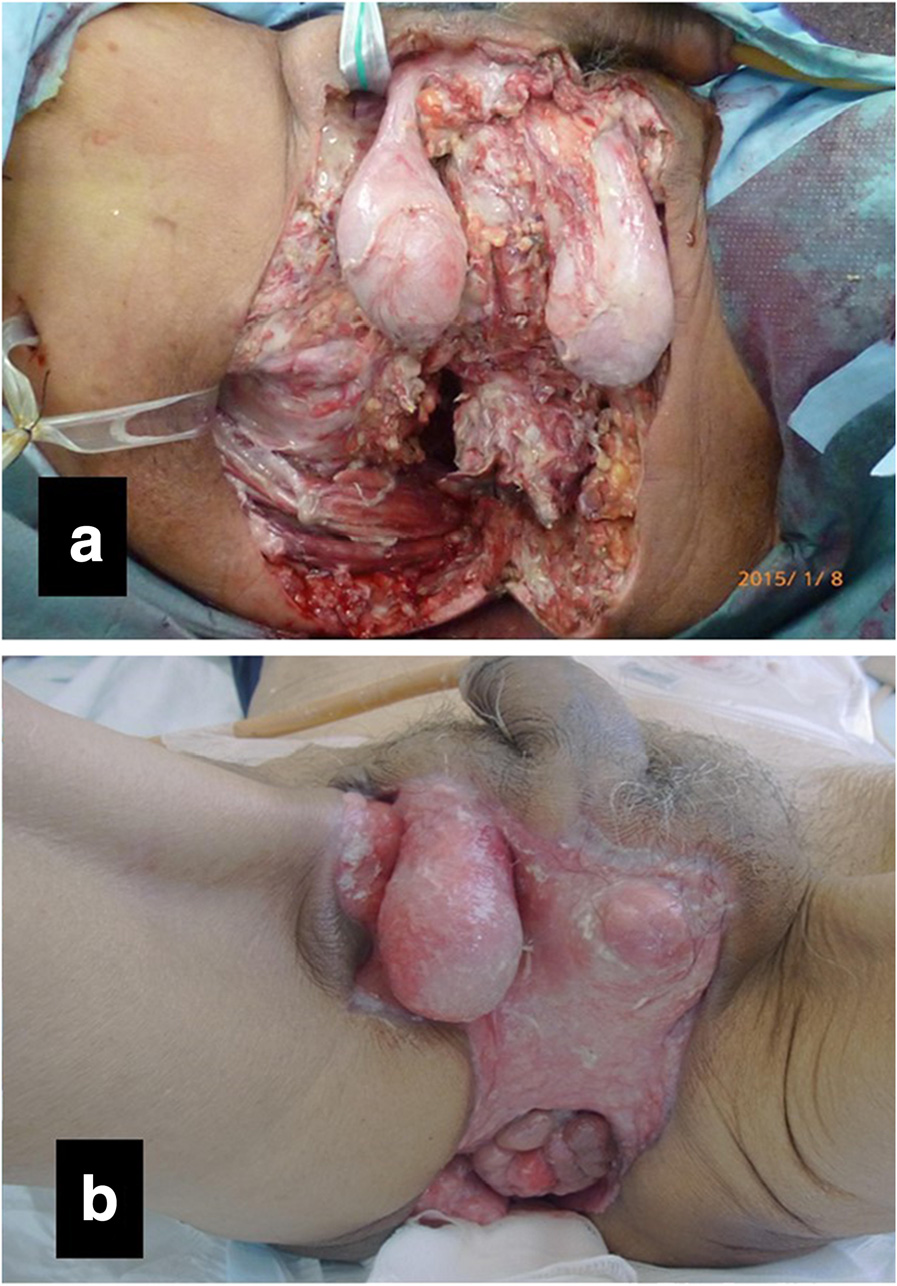
Extensive debridement and diverting colostomy were performed on the day of admission (**a**). Fifty days later, a more extensive debridement was performed (**b**)

A 65-year-old man was brought to our outpatient hospital in an ambulance complaining of general fatigue and anal pain. The medical and surgical histories were unremarkable. His initial body temperature was 95.7 °F, blood pressure was 125/58 mmHg, heart rate was 92 beats/min, and oxygen saturation was 98 % on room air. On physical examination, a black spot on the perineal skin surrounded by erythema was found. Due to a clinical suspicion for FG, a computed tomography (CT) scan and blood tests were obtained urgently (Fig. 1). CT scan showed abscess formation with emphysema around the rectum, as well as a tumor invading the bladder (cT4b) with some lymph node metastases in the mesorectum (cN2); there was no evidence of distant metastasis (M0) (Fig. 2). Blood tests were remarkable for a hemoglobin of 3.2 g/dl and a leukocytosis of 63,200/μl. The patient was thus diagnosed with locally advanced rectal cancer and FG with a severity index score of 12 points [1]. Urgent diverting colostomy of the transverse colon and extensive debridement of the perineal area were performed (Fig. 3). Biopsies taken previously revealed a moderately differentiated adenocarcinoma.

**Fig. 4:**
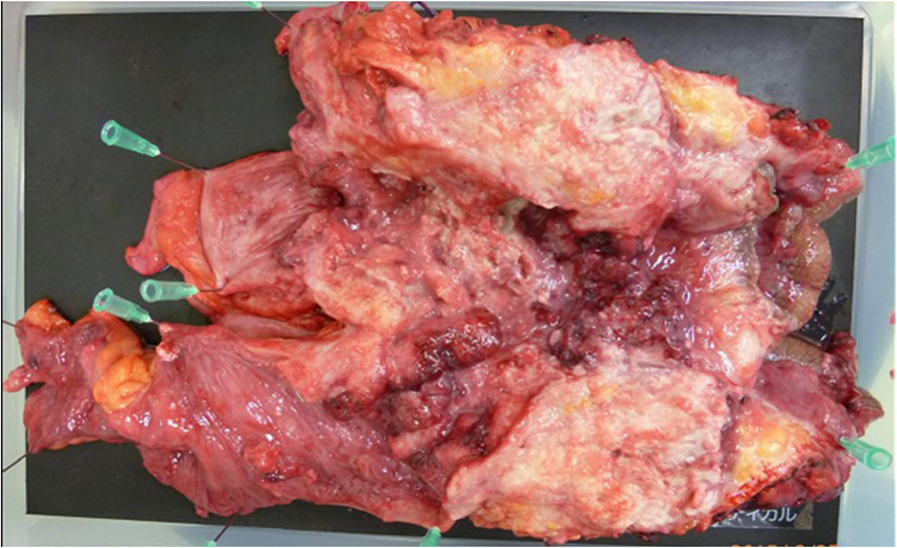
TPE with sacrectomy was performed to obtain a negative resection margin. Histopathological findings of the specimen revealed a moderate adenocarcinoma invading into the bladder and the prostate (T4b), metastasis to four perirectal lymph nodes (N2), and lymphovascular invasion

**Fig. 5:**
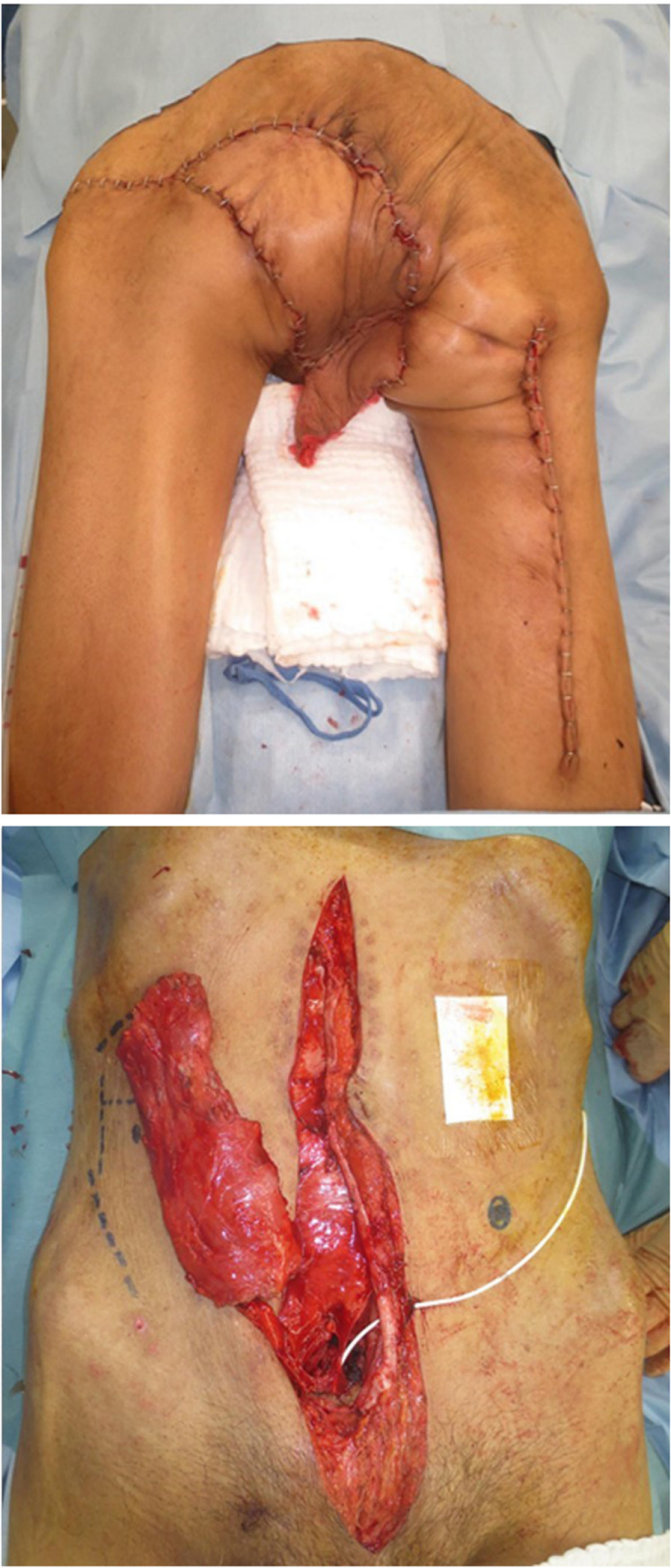
Reconstruction was performed using the rectus muscle, the gluteus maximus muscle, and the femoral muscle

The patient received nutritional support after surgery. Follow-up CT scan after the surgery showed no new lesions, so we performed a radical total pelvic exenteration (TPE) with sacrectomy 50 days after the initial surgery. For the urinary diversion, Bricker’s operation was performed (Fig. 4). In addition, reconstruction of the soft tissue defect was performed using the rectus muscle, the gluteus maximus muscle, and the femoral muscle (Fig. 5). Histopathological findings of the specimen were as follows: the tumor was a moderate adenocarcinoma with invasion to the bladder and the prostate (T4b), metastases to four resected lymph nodes (N2), and lymphovascular invasion. There were no major postoperative complications, and the patient was discharged on postoperative day 108.

### Discussion

Although perforation of rectal cancer after treatment with bevacizumab or radiation therapy has been well documented, reports of spontaneous perforation of rectal cancer presenting as FG are rare. There were 23 cases evaluated in a review by Bruketa et al. [2]; in Japan, 17 cases including the present case have been reported (Table 1). The median age in the Japanese group was 58 (range, 30–80) years with a male to female ratio of 15:2. Six (35 %) of 17 patients had diabetes mellitus (DM) as a comorbidity. DM was considered a risk factor for FG in the Japanese group as well. Colostomy was created in 9 of 17 patients. Treatment for rectal cancer with FG was abdominoperineal resection (APR) for six patients, TPE for three patients, and chemotherapy alone for three patients. Four patients were followed conservatively without treatment. Regarding long-term prognosis of the patients, survival of 4 years and 7 months was obtained in one patient who underwent APR, and the survival of the other 16 patients was not given. In this patient, we performed TPE with sacrectomy to obtain a negative resection margin, resulting in a good oncological outcome. In addition, reconstruction was performed using the rectus muscle, the gluteus maximus muscles, and the femoral muscle. There were no major postoperative complications, and the patient was discharged on postoperative day 108.

**Table 1:** Case reports of Fournier’s gangrene in the setting of rectal cancer in Japan

No.	Author		Gender	Age	Location	Comorbidity	Operation	Outcome
1	Futamura et al. [6]	1995	M	56	Rb	None	APR	4 years and 7 months, alive
2	Fujisawa et al. [7]	1999	M	75	Rb	None	None	Death at 6 days
3	Nakao et al. [8]	1999	M	51	Rb	DM	None	Unknown
4	Saito et al. [9]	2000	M	60	Rb	None	TPE	1 year, alive
5	Noriyuki et al. [10]	2003	M	58	Ra	DM	5-FU + LV	1 year and 2 months, alive
6	Enomoto at al. [11]	2006	M	35	Rb	None	APR	3 months, alive
7	Moriwaki et al. [12]	2007	M	30	Rb-P	Unknown	APR	Death at 11 months
8	Kojima et al. [13]	2007	M	56	Rb	DM	TPE	4 months, alive
9	Morohashi et al. [14]	2008	M	60	Rb	DM	None	Death at 2 months
10	Ishibashi et al. [15]	2009	F	80	Rb	HT	APR	2 months, alive
11	Yamazaki et al. [16]	2010	M	50s	Rb	DM	APR	1 months, alive
12	Onizuka et al. [17]	2010	M	55	Rs	None	Chemo	1 year and 2 months, alive
13	Tanaka et al. [18]	2010	F	52	Rb	DM	APR	5 months, alive
14	Watabe et al. [19]	2013	M	77	Rb-P	Unknown	None	2 months, alive
15	Monma et al. [20]	2013	M	79	P	HT	CRT	1 year, alive
16	Kawagoe et al. [21]	2014	M	72	Rb	None	FOLFOX + bev	6 months, alive
17	Our case	2016	M	65	Rb	None	TPE	Death at 1 year

*M* male, *F* female, *Rb* lower rectum, *Rs* rectosigmoid colon, *P* proctos, *APR* abdominoperineal resection, *TPE* total pelvic exenteration, *Chemo* chemotherapy, *CRT* chemoradiation therapy, *Bev* bevacizumab, *DM* diabetes mellitus, *HT* hypertension

**Fig. 6:**
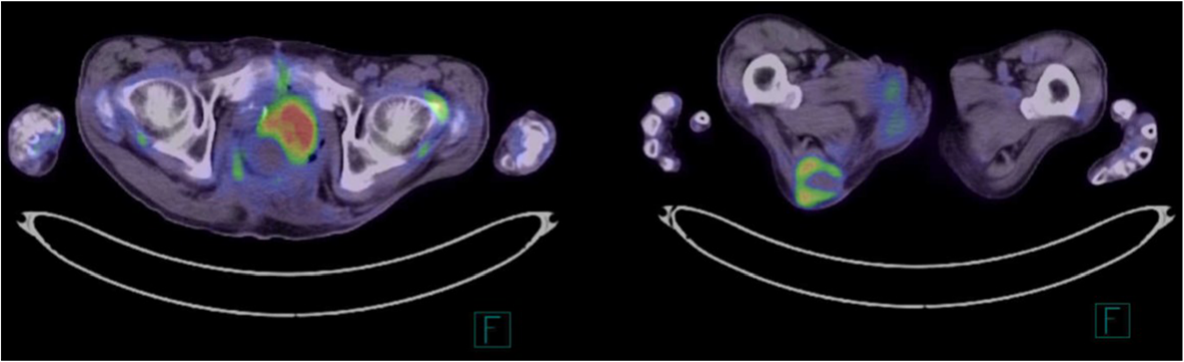
^18^F-fluorodeoxyglucose positron emission tomography scan demonstrated increased fluorodeoxyglucose uptake lesions in the perineal wound. The maximum standardized uptake values (SUV max) of the lesions were 6.36 and 13.45, respectively. Local recurrence was suspected because of an abnormal ^18^F-fluorodeoxyglucose positron emission tomography scan

The mortality of FG is high, frequently due to a delay in the diagnosis and management. The interesting report about the metabolic evaluation of FG was reported by Surucu et al. [3]. They showed the significance of ^18^F-fluorodeoxyglucose positron emission tomography for FG in metabolic levels before the visual changes occurred. The mortality of FG in the setting of rectal cancer is not clear. The most common causes of death in FG are sepsis and multiple organ failure. Prognostic predictions can be based on Fournier’s gangrene severity index (FGSI), comprised of the following: body temperature, heart rate, respiratory rate, and serum level of sodium, potassium, creatinine, and bicarbonate, as well as hematocrit value and leukocyte count [1]. Laor et al. [1] stated that a score of >9 was associated with a 75 % mortality, while a score of ≤9 corresponded to a 78 % probability of survival. While our patient presented with an FGSI score of 12 points, we managed to treat the patient without a major complication. Recently, hyperbaric oxygen (HBO) has been recommended as an additional therapy for patients with FG because HBO inhibits the growth of anaerobic bacteria in the affected tissues, prevents further extension of tissue necrosis, and reduces systemic toxicity [4, 5]. Actually, for this patient creating colostomy as an initial procedure, early radical debridement of necrotic tissues, drainage, and antibiotic therapy were effective to manage severe FG in the setting of rectal cancer. Although TPE with sacrectomy was required to obtain a negative resection margin, we could get a good outcome. Although adjuvant therapy was required because of locally advanced rectal cancer of pathologically T4b N2 M0, the patient was followed up at the outpatient section of our institution without adjuvant therapy until all wound had healed completely. Unfortunately, we suspected that local recurrence developed in the perineal wound because of an abdominal ^18^F-fluorodeoxyglucose positron emission tomography in 5 months after curative TPE (Fig. 6). The patient died of primary disease 1 year after prior surgery.

## Conclusions

Aggressive debridement, broad-spectrum antibiotics, and intensive supportive care are critical for the management of FG. Although colorectal cancer has previously been reported as a cause of FG, it remains an extremely rare phenomenon.

## Abbreviations

FG: Fournier’s gangrene
CT: Computed tomography
TPE: Total pelvic exenteration
APR: Abdominoperineal resection
FGSI: Fournier’s gangrene severity index
